# *Lactococcus lactis*, an Attractive Cell Factory for the Expression of Functional Membrane Proteins

**DOI:** 10.3390/biom12020180

**Published:** 2022-01-22

**Authors:** Annie Frelet-Barrand

**Affiliations:** FEMTO-ST Institute, UMR 6174, CNRS, Université Bourgogne Franche-Comté, 15B Avenue des Montboucons, CEDEX, 25030 Besançon, France; annie.frelet-barrand@femto-st.fr

**Keywords:** *Lactococcus lactis*, membrane proteins, NICE system

## Abstract

Membrane proteins play key roles in most crucial cellular processes, ranging from cell-to-cell communication to signaling processes. Despite recent improvements, the expression of functionally folded membrane proteins in sufficient amounts for functional and structural characterization remains a challenge. Indeed, it is still difficult to predict whether a protein can be overproduced in a functional state in some expression system(s), though studies of high-throughput screens have been published in recent years. Prokaryotic expression systems present several advantages over eukaryotic ones. Among them, *Lactococcus lactis* (*L. lactis*) has emerged in the last two decades as a good alternative expression system to E. coli. The purpose of this chapter is to describe *L. lactis* and its tightly inducible system, NICE, for the effective expression of membrane proteins from both prokaryotic and eukaryotic origins.

## 1. Introduction

Membrane proteins (MPs), key proteins in cell physiology and drug targets, are encoded by one-third of the human genome [1,2]. MPs have different features: (i) they form various topologies, from peripheral to intrinsic polytopic proteins with a high number of transmembrane helices, (ii) their surface is relatively hydrophobic, (iii) detergents are required for their solubilization from the cell membrane, and they often need to be reconstituted into proteoliposomes for functional studies, (iv) they are flexible and unstable, (v) they must be targeted to the membrane for proper folding, (vi) they are expressed at very low levels, and/or (vii) they are functional in homo- and hetero-oligomeric states [3,4]. In order to increase and deepen our knowledge, in particular for pharmaceutical purposes, there is an increasing need for structural and functional studies [5]. During the last 7 years, the number of unique 3D structures of MPs increased from 400 to 1348 (https://blanco.biomol.uci.edu/mpstruc/, accessed on 21 December 2021), which is still far away from the 75,000 structures available for soluble proteins. The reason why the number of 3D structures is still so low is linked to the difficulty of obtaining sufficient amounts of functionally folded MPs. Functional and structural studies require high amounts of proteins. Therefore, the low concentration of MPs in cells highlights the need for heterologous expression systems. There are different types of expression systems such as cell-free systems [6], prokaryotic systems (*E. coli* and *L. lactis*), and eukaryotic expression systems (yeasts, plants, mammalian, or insect cells). All of them have advantages and drawbacks [3,4,7]. Bacteria are the most used systems for the expression of recombinant proteins, including MPs and the first hosts used prior to the other expression systems listed above, because they are easy to handle and inexpensive compared to eukaryotic systems. Furthermore, a wide range of genetic methods and vector systems are well-established. Among them, *E. coli* can be considered as the traditional and oldest bacterial gene expression system, which has been developed for many years and whose wide variety of plasmids and host strains are available. In most cases, the induction of gene expression is based on IPTG (IsoPropyl β-D-1-ThioGalactopyranoside) [8,9]. However, the yield of functional MPs is often unsatisfactory, which is generally due to the formation of inclusion bodies, the production of endotoxins and proteases by the bacteria, and/or the high translation rate [9,10]. In the last twenty years, another bacterium emerged as a good alternative to *E. coli* for the expression of MPs, i.e., *Lactococcus lactis*.

## 2. *Lactococcus lactis*

*Lactococcus lactis*, a Gram-positive bacterium, emerged at the beginning of the twenty-first century as a good alternative to the functional expression of prokaryotic and eukaryotic MPs [7,11,12]. This bacterium grows at 30 °C, with a doubling time of 35–60 min, and grows with a fermentative or respiration type of metabolism [13]. Although largely used in the food industry for the production of fermented foods, its potential as a host for the overexpression of homologous and heterologous proteins has also been explored [14,15,16]. *L. lactis* is easy and inexpensive to grow, a large variety of genetic methods and vector systems are available and well-developed. Therefore, *L. lactis* is an interesting alternative gene-expression host, especially for eukaryotic MPs, because of its moderate proteolytic activity, the absence of inclusion-body formation and of endotoxin production, and its efficient targeting of the MPs into a single plasma membrane [11,17,18]. The absence of endotoxin allows for the use of the bacteria or the protein produced by the bacteria for biotechnological and therapeutic applications [14,16]. *L. lactis* does not form inclusion bodies since other factors/mechanisms take place: mRNAs of recalcitrant MPs form polar clusters, leading to the cessation of cell division and to degradation rather than aggregation [19].

Moreover, this bacterium allows for the performance of functional studies directly with intact cells and membrane vesicles [11,20]. *L. lactis* possesses different lipids within the membrane and is particularly rich in glycolipids and cardiolipin, lipids not present in *E. coli* membranes [21,22]. Lipids are essential for MPs, for its stability, conformations, and functionality; depending on the nature and functions of the MP produced, this specific lipid composition could have a positive influence on the expression and the functionality of the MP [3,4,23]. In this review, examples of successful MP expressions linked to the *L. lactis* lipid composition will be described (see Section 4. Functional expression of MPs), particularly the relationship between MPs from the AAC family and cardiolipin and MPs from chloroplasts and glycolipids.

*L. lactis* has a genome half the size of that of *E. coli* and may lack specific chaperone systems and other auxiliary factors which could be necessary for the targeting and correct folding of particular MPs [11]. Its codon usage is an approximative 65% biased for AT base pairs. Therefore, the gene encoding the protein of interest needs to be optimized for the codon usage in *L. lactis* [20]. One difficulty of working with *L. lactis* is in the cloning efficiency [24]. Hence, in order to facilitate and obtain a larger number of recombinant clones, different strategies have been developed in the last years in addition to the classical one (see Section 2.3.2. New cloning strategies).

The expression of heterologous proteins in *L. lactis* has been facilitated by the advances in genetic knowledge and new developments in molecular biology techniques. Using these tools, various vectors containing either constitutive or inducible promoters have been developed to obtain increased levels of proteins and control their production. They currently constitute the basis of all expression systems in *L. lactis* and other lactic acid bacteria [25]. Among the various expression systems, the NICE system represents the most used system for proteins, particularly MPs, in *L. lactis* [26]; different strains of this bacterium have been optimized for MP expression (see Section 2.2. Host strains used for NICE system).

### 2.1. The Nisin-Controlled Gene Expression System (NICE)

**Figure 1:**
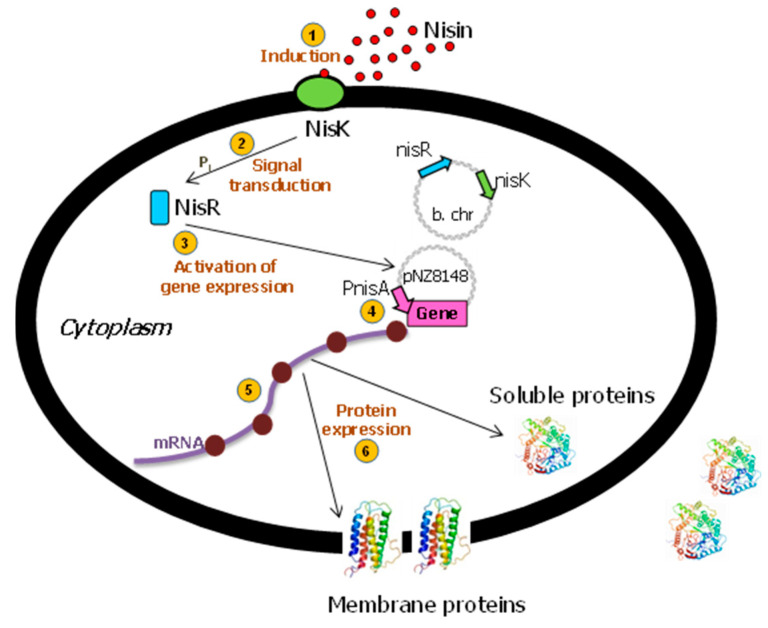
The Nisin-Controlled Gene Expression (NICE) system in *L. lactis*. After the detection of nisin by the membrane-located sensor protein (NisK) ①, this histidine protein kinase autophosphorylates and transfers its phosphate group to activate the cytoplasmic response regulator NisR ②. Activated NisR ③ subsequently induces transcription controlled by the PnisA promoter ④. After transduction, ⑤ and depending on the presence or absence of the corresponding targeting signals, the protein is either expressed into the cytoplasm or the membrane, or secreted into the external medium ⑥. B. chr: bacterial chromosome. Adapted from [12,25].

The tightly regulated NICE (Nisin-Controlled Gene Expression) system is the most broadly and commonly used gene expression system in *L. lactis* [16,20]. This promising and effective expression system was developed for lactic acid bacteria and is based on genes involved in the biosynthesis and regulation of the antimicrobial peptide, nisin (product of the nisA gene). This 34-amino-acid bacteriocin produced by several strains of *L. lactis* [26] can also be used as a natural food preservative [27]. The genes of the two-component signal transduction system, nisK and nisR, from the nisin gene cluster were inserted into the chromosome of *L. lactis* subsp. cremoris MG1363 (nisin-negative) [28] to create the strain NZ9000 [29,30]. When a gene of interest is subsequently placed behind the inducible promoter PnisA in a plasmid [31], the expression of that gene can be induced by the addition of sub-inhibitory amounts of nisin (0.1–5 ng/mL) to the culture medium [32] (Figure 1), either obtained commercially or by adding the supernatant from the NZ9700 nisin-secreting lactococcal strain. In order to obtain higher yields, the growth medium, fermentation conditions, and nisin induction were optimized [14].

Well-characterized and highly versatile, the NICE system has been widely used for the over-expression and the subsequent functional and structural studies of homologous and heterologous MPs [12]. Moreover, it has been used for other purposes such as in pharmaceutical, medical, biotechnology, and food-technology applications [15,16,33]. Recently, the NICE system has been combined with the ZIREX system, allowing for the expression of different proteins at different times during the growth cycle [34].

Moreover, this NICE system has also been transferred to other Gram^+^ bacteria (*Leuconostoc lactis*, *Lactobacillus brevis*, *Lactobacillus helveticus*, *Lactobacillus plantarum*, *Streptococcus pyogenes*, *Streptococcus agalactiae*, *Streptococcus pneumoniae*, *Streptococcus zooepidemicus*, *Enterococcus faecalis,* and *Bacillus subtilis*) but without use for the high-scale production of MPs. Indeed, in many cases, regulated gene expression was established, but the growth of several species is retarded by the introduction of a special dual plasmid system, the different nisin sensitivity presented by the strains, the RNA polymerase sequence, and other factors influencing the expression of MPs [33].

In addition, the NICE system presents some drawbacks [20,33]: the maximum cell density obtained in a normal simple M17 acidifying buffered culture is about OD600 = 3 (1 g/L dry cell mass), lower than the density obtained from aerobic strains (100 g/L). The growth stops at a pH of around 5. Different techniques can be used to increase the cell density, including neutralization with NaOH or NH_4_OH, resulting in a maximum of OD600 = 15 (5 g/L dry cell mass), as well as growth under aerobic conditions in the presence of haem. Additionally, the proteolytic degradation of heterologous proteins is also a limiting factor in stable protein production; one recombinant strain has been constructed with the inactivation of the single protease HtrA, in which protein degradation is lower.

### 2.2. Host Strains Used for the NICE System

Different *L. lactis* host strains derived from *L. lactis* subsp. cremoris MG1363 can be used for the expression of cDNAs with the NICE system (Table 1, [28]). The most commonly used host strain for MP expression is the strain NZ9000. The nisin-producing strain NZ9700 [28] was obtained by the conjugation of the nisin–sucrose transposon Tn5276 of the nisin-A producer NIZO B8 with MG1464, a rifampicin- and streptomycin-resistant derivative of MG1363 [34]. Since the expression of MPs in *L. lactis* encounters difficulties due to low expression yields, different strategies have been developed to enhance their production. These strategies are either based on the introduction of an N-terminal fusion protein [19], mutations in the NisK ATPase domain of the sensor kinase (R406C) resulting in the DML1 strain [35], inactivation of the unique protease HtrA [36], selection of a strain enabling a higher plasmid stability (M4; [37]), or the overexpression of the cell envelope stress sensor/regulator CesSR [38].

### 2.3. cDNA Cloning in Expression Vectors

#### 2.3.1. Classical Cloning Using Restriction Enzymes

The cDNA, or the gene encoding the MP of interest, is cloned into the appropriate expression plasmid, i.e., pNZ8048 or its derivatives (Table 1). These plasmids are based on the pSH71 replicon carrying the chloramphenicol resistance gene [31]. Plasmid pNZ8048 is the most commonly used plasmid for translational fusions. Genes of interest are directly fused to the *Nco*I site, which contains the ATG start codon directly downstream of the PnisA promoter. Different variants of pNZ8048 have been constructed: pNZ8148 is a shorter version of pNZ8048, with the deletion of a 60 bp heterologous DNA fragment from *Bacillus subtilis*, the initial cloning host of the pSH plasmid series [40]. pNZ8150 possesses a *Sca*I site directly upstream of the ATG start codon and therefore avoids the obligatory use of the *Nco*I site. Thus, it is no longer necessary to change the second amino acid of a protein if that codon does not conform with the sequence of the *Nco*I site. Other plasmids and strains are available and can be used for other purposes [20,33] (Mobitec Molecular Biotechnology; https://www.mobitec.com, accessed on 21 December 2021). The unidirectional cloning using classical restriction enzymes allows for a higher number of recombinant clones after transformation. Nevertheless, the MCS site is relatively small, containing less than 10 restriction sites, and partial digestions or mutagenesis is often required to obtain the desired constructs.

#### 2.3.2. New Cloning Strategies

In addition to the classical cloning approaches, new strategies have been developed to overcome the problem of the low efficiency of gene manipulation in *L. lactis* and the instability of *L. lactis*–*E. coli* shuttle vectors [41,42]. Examples are ligation-independent cloning (LIC) and Gateway and other technologies developed by Berlec and collaborators. Furthermore, Geertsma and Poolman developed a generic cloning strategy compatible with high-throughput manipulations, which is also suitable for organisms other than *L. lactis* [43]. This method involves ligation-independent cloning (LIC) in an intermediary *E. coli* vector (pRExLIC-geneX), which can rapidly be converted via vector-backbone exchange (VBEx) into an organism-specific plasmid that is ready for high-efficiency transformation, such as pNZxLIC-geneX for *L. lactis*. In both LIC and VBEx procedures, rare restriction sites (*Swa*I and *Sfi*I) were used. This strategy allowed for the successful expression of MPs from prokaryotic and eukaryotic origins [44,45,46].

Other laboratories developed strategies based on the Gateway technology (Invitrogen), which are now widely used to simplify the cloning of cDNAs into many different expression systems, from bacteria to eukaryotic systems [47], and for the high-throughput expression screening of integral MPs [48]. Several libraries are currently available in Gateway-compatible vectors [49]. However, *L. lactis* plasmids (e.g., pNZ8048 or derivatives) cannot be converted into Gateway destination vectors. Therefore, a strategy for the preservation of the correct reading frame has then been established for the rapid transfer of the cDNA from Gateway entry vectors into *L. lactis* nisin-inducible vectors [12,50]. This strategy allows for the successful expression of MPs from prokaryotic and eukaryotic origins, including proteins which could not be expressed using traditional cloning [7,51]. Only one development using an *E. coli*-*L. lactis* shuttle vector containing the Gateway cassette was proposed. These vectors allowed for the expression of two lactococcal phages, Tuc2009 and TP901-1 [52], and methyltransferases [53], but not of MPs.

Furthermore, in order to obtain a higher number of insert-containing plasmids after transformation, Berlec and Strukelj [54] developed a TA-cloning expression plasmid. A few years later, Berlec developed pNZ vectors for the dual expression of the proteins pNZDual and pNZDualTT, and one additional vector for the expression of proteins from polycistronic RNAs, pNZPolycist [55]. For the combinations tested, expression was higher using the latter compared to the pNZDual versions. Only one article showed the dual expression of secreted proteins fused to the usp45 secretion signal [56]. This point needs to be further investigated with different combinations of MPs to verify the impact of such constructs on the expression of MPs.

Once a gene is cloned within the proper vector, recombinant bacteria could be generated and used for MP expression through the NICE system.

## 3. Expression of Membrane Proteins Using the NICE System

In the last twenty years, the NICE system has proved to be highly versatile for the expression of proteins, including MPs using pNZ8048 and its derivatives. Up to now, 113 MPs from prokaryotic or eukaryotic origin, with diverse topologies and sizes, have been successfully expressed, including 79 in 2014 (12 and the present, Table 2, Table 3 and Table 4). This system also allows for the expression of MPs in their native oligomeric form (homo or heterodimers) [11,12].

Table 2, Table 3 and Table 4: List of homologous and heterologous prokaryotic and eukaryotic MPs expressed in *L. lactis* using the NICE system. Species, size, expression levels, and functions are given for each protein; the classification of MPs has been sorted according to the protein complexity in terms of numbers of TM helices. UNIPROT (http://www.uniprot.org/, accessed on 21 December 2021) was used as reference for protein information in addition to the literature.

**Figure 2:**
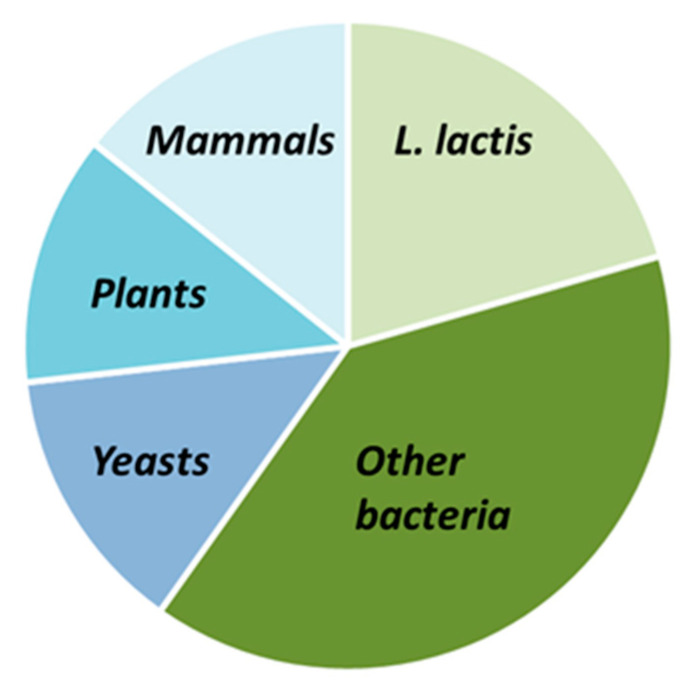
Comparison of MPs expressed in *L. lactis* using the NICE system depending on their origin: *L. lactis*, other bacteria, or eukaryotic cells.

Table 2, Table 3 and Table 4 display respectively non-exhaustive lists of prokaryotic MPs (homologous or heterologous expression) and eukaryotic MPs expressed in *L. lactis* with the NICE system. They include studies of functionally active proteins in which expression levels were not determined. The tables do not display, for some proteins, the percentage of expressed proteins when not available, and that of functional proteins out of the proteins expressed; indeed, this information is seldom reported since such a ratio is difficult to measure and necessitates isolating native proteins as controls. *L. lactis* MPs represent 20% of the total number of MPs expressed, while prokaryotic MPs are at 40%, and eukaryotic ones comprise 40%, respectively; among the latter, each origin (yeast, plant, and human) represents one-third (Figure 2).

**Figure 3:**
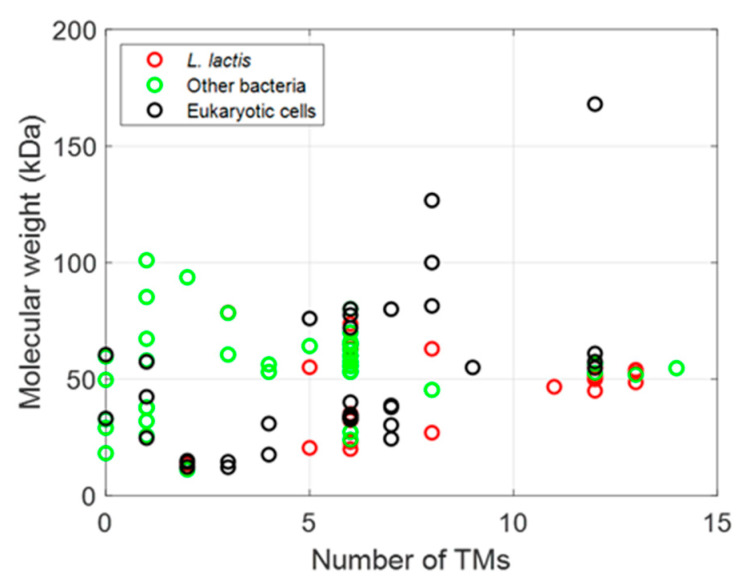
Influence and relationship between origins of MPs expressed in *L. lactis*.

**Figure 4:**
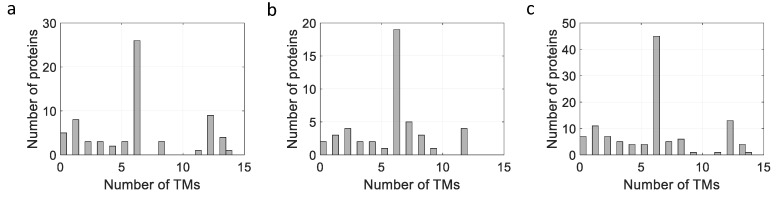
Influence of number of TM helices on expression of MPs expressed in *L. lactis*. (**a**) On expression of MPs from *L. lactis*. (**b**) On expression of MPs from other bacteria. (**c**) On expression of eukaryotic MPs.

The membrane proteins listed in Table 2, Table 3 and Table 4 can be plotted according to the number of their TM helices and their molecular sizes. As shown in Figure 3, a large number of MPs have sizes below 100 kDa, with many MPs having either 6 or 12 TM helices, whether they are prokaryotic, from *L. lactis* or other bacteria, or are of eukaryotic origin (Figure 3 and Figure 4), thus highlighting the two large families of proteins expressed (ABC and mitochondrial transporters).

### 3.1. Expression of Prokaryotic MPs

Table 2 and Table 3 report the successful expressions of 23 homologous and 43 heterologous MPs using the NICE system. The expression levels of prokaryotic MPs obtained were the highest of all reviewed MPs, with up to 30% of total MPs (TMP) by heterologous (HorA and MsbA) and homologous (LmrA) expression. The expressed MPs possess up to 13 TM helices, and even with such a high TM helix content, they were produced with expression levels of up to 20% TMP (BcaP and XylP). Most homologous MP expression studies have been focused on proteins belonging to the families of amino acid and ABC (ATP-Binding Cassette) transporters, probably related to the specialization of the laboratories working with this system. In addition to the above-mentioned amino acid and ABC transporters, other heterologous MPs have been expressed, belonging to diverse families such as cytochrome, permease, and binding proteins (Table 3). The relatively high expression levels obtained with heterologous prokaryotic MPs could be explained by the fact that the codon usage is compatible with the AT-rich codon bias of *L. lactis* [107]. *L. lactis* also allowed for the expression of an MP with 14 TM domains, such as the MFS transporter called Rv1410 (Table 3; [85]).

### 3.2. Expression of Eukaryotic MPs

The expression of eukaryotic MPs in *L. lactis* was initiated and first reported in 2003 by Kunji and collaborators, together with the expression of mitochondrial carriers from yeast [11]. Since then, several other eukaryotic MPs from yeast, plants, and humans have been expressed, with levels from 0.1 to 10% of TMPs (Table 4), mainly from the mitochondrial carrier superfamily, but also from other families. Only one MP from protozoa (*A. polyphaga*; Table 4; [100]) was expressed in *L. lactis*.

#### 3.2.1. Membrane Proteins from Yeast (*S. cerevisiae*)

A total of 16 MPs from yeast have been successfully expressed in *L. lactis*. Two main studies on mitochondrial carriers revealed that all the MPs tested could be expressed with levels from 0.5 to 10% (Table 4). For some of them, expression levels were even improved by the rational design of the N-terminus (replacing or truncating these regions or by adding lactococcal signal peptides) [18].

#### 3.2.2. Membrane Proteins from Plants

A total of 13 MPs from three plant species, i.e., *A. thaliana, S. tuberosum,* and *N. patriciarum*, have been successfully expressed in *L. lactis*. They belong to different families, for instance, an oxidase and various transport proteins (heavy metal, ATP/ADP, or sucrose), and their topologies span from peripheral to intrinsic 12 TM helices (Table 4). The levels of expression obtained were relatively high, up to 30% (Table 4), without modifications of the sequence. These relatively high expression levels allowed for the performance of functional studies to discover and/or go deeper into the function of the MP expressed.

#### 3.2.3. Membrane Proteins from Humans

As for yeast mitochondrial carriers, human ADP/ATP translocators (AAC1, AAC2, and AAC3) were also expressed in *L. lactis*. Other human MPs from diverse families and topologies (1–12 TM helices) have been expressed, with levels from almost undetectable (<0.1%) to 1% (Bcl-Xl) (Table 4), including the ABC transporter, CFTR with a very high number of TM helices (12 helices), and size (168 kDa) expressed at very low levels (below 0.1% of TMP; [46]).

### 3.3. Comparison of Expression Levels between E. coli and L. lactis

The expression levels obtained for the expression of MPs in *L. lactis* are generally lower than those obtained for the overexpression of the same MPs in *E. coli* [7,12,60]. In some cases, the expression in *L. lactis* allowed for a higher expression or the expression of proteins usually produced in inclusion bodies in *E. coli*. For proteins produced with both bacterial expression systems, the levels were almost 10 times lower after expression in *L. lactis* as compared to that in *E. coli* [12]. The mechanisms for the production of proteins in both bacteria are different, and inclusion body formation allows *E. coli* to produce higher amounts of protein in inclusion bodies, mostly in-correctly folded or mis-folded, and therefore not functional. The slower protein synthesis is an advantage for *L. lactis* since it leads to the proper and correct functional folding acquisition of the MP produced in a single membrane. This slowness could be due to a limitation of amino acid import, especially for branched amino acids. This problem could be overcome by supplying the cells with an alternative path, such as a medium containing the appropriate dipeptides or by engineering the transport capacity of branched-chain amino acids [108]. Other strategies have been implemented using the optimization of functional expression, i.e., control of transcription rate, nutrient availability in richer medium, gene optimization, and/or fusion tags [60].

All MPs listed in Table 2, Table 3 and Table 4 have been expressed in *L. lactis* and were functional in this bacterium, which allowed for different assays to be performed and to decipher/discover the function of the MPs in the original organism.

## 4. Functional Expression of MPs

**Figure 5:**
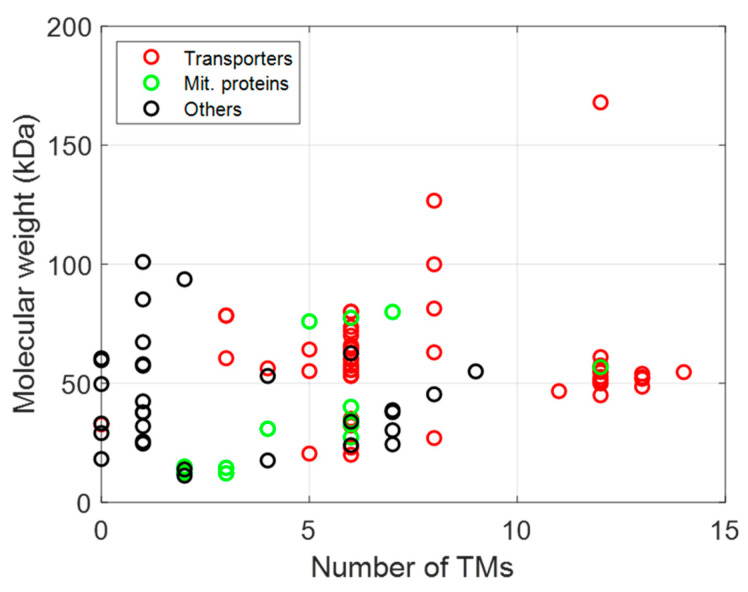
Relationship between function (transporters in red, mitochondrial proteins in green, and other functions in black), size, and topology of MPs expressed in *L. lactis*.

The following section will focus on examples of MPs of either prokaryotic or eukaryotic origin belonging to one functional class such as ABC transporters, secondary transporters, etc. *L. lactis* presents three major advantages over *E. coli* for functional MP expression: (i) it possesses only one membrane; (ii) it does not form inclusion bodies, and (iii) it expresses proteins in their native oligomeric state. Moreover, the genomes of MG1363 and NZ9000 are completely sequenced and annotated, allowing for the generation of mutated strains. These functional characterizations could be performed on: (i) whole bacteria using radioactive substrates, (ii) membrane vesicles, (iii) proteoliposomes after reconstitution with phospholipids, and/or (iv) solubilized/purified proteins. All MPs expressed (Table 2, Table 3 and Table 4) belong to different families: ABC transporters, secondary transporters, MPs originating from organelle (mitochondria, chloroplast), MCP, and other families (Figure 5).

### 4.1. ABC Transporters

ABC transporters generally consist of four domains—two membrane-embedded domains carrying out substrate recognition and translocation and two hydrophilic nucleotide binding domains (NBDs). They represent one-third of the MPs expressed in *L. lactis* (Table 2, Table 3 and Table 4; Figure 5). Either transport or ATPase activities can be measured with radioactive or non-radioactive compounds on intact cells or detergent-purified protein within or outside of proteoliposomes or nanodiscs. In some cases, mutations allowed for the role assignment of certain amino acids to the proper function of the proteins. Studies in intact cells were facilitated by the availability of strains deleted in LmrACD, the three main ABC transporters present in the *L. lactis* membrane.

The ABC half-transporter LmrA (65 kDa, six TM helices), a well-characterized ABC transporter from *L. lactis,* was expressed in very high levels (up to 30% of TMP; [64]). The critical role of a carboxylate group in proton conduction to secondary-active transporters could be assigned [109]. Additional studies were performed on mutated versions expressed in *L. lactis* wild-type strains or strains with a deletion of LmrA homologs (LmrCD) [65]. Different studies based on nuclear magnetic resonance (NMR) and electron spin resonance (EPR) spectroscopy allowed for an understanding of the ATP hydrolysis cycle of the protein, the nucleotide binding, and the induction of the ion-motive force [110,111,112,113].

The thiamine high-affinity ABC transporter, ThiT (20 kDa, six TM helices), which belongs to the family of energy coupling factors, has been characterized in *L. lactis*. The expression levels in *L. lactis* was around 1–2% (Table 2; [45]). Mutagenesis studies allowed for the determination of some amino acids interacting with the energizing module, necessary for vitamin translocation [114]. EPR performed on purified ThiT and molecular dynamic studies allowed for a detailed description of the conformational changes of the protein during binding and coupling with the energizing module [83]. The structure of this protein was solved in 2014 [115].

Moreover, out of the 31 ABC transporters that have been expressed in *L. lactis*, 19 originated from other bacteria. Among them, the half ABC-transporter MsbA from *E. coli* was expressed with a level slightly lower than that obtained from the homologous expression of LmrA (20–30%). This homodimeric transporter, with six TM helices and a molecular size of 64 kDa, is involved in lipid A export in *E. coli* [78]. Functional studies have demonstrated that substrate binding to the MsbA dimer caused NBD dimerization [116,117,118].

A heterodimeric ABC exporter, TM287/288 from *Thermotoga maritima*, has also been expressed in *L. lactis* [91]. TM287 and TM288, with a molecular size of 60 kDa and six TM helices each, form a functional heterodimer that shares 36% of its sequence identity with LmrCD, a well-characterized heterodimeric ABC exporter from *L. lactis* [65]. Functional studies determined that the NBDs only partially separate and remain in contact through an interface involving conserved motifs connecting the two ATP hydrolysis sites [91].

Finally, some eukaryotic ABC transporters were expressed in *L. lactis*. Among them, the well-known CFTR [42] and a plant mitochondrial ABC transporter, ATM3/ABCB25. Membrane vesicle assays revealed that glutathione (GSH) polysulfides are likely to be the substrates serving as precursors for iron-sulfur cluster assembly [92].

### 4.2. Secondary Transporters

Secondary active transporters exploit the electrochemical potential of solutes to shuttle specific substrate molecules across biological membranes, usually against their concentration gradient. These proteins are involved in the transport of amino acids [119], organic or inorganic anions, through symport or exchange processes [120]. MPs from the MFS superfamily were successfully expressed in *L. lactis* in their functional state [119,120]. Whilst the quantity of proteins produced in these studies was not determined, the biological activity of the proteins was detected using substrates specific to the transporters.

### 4.3. MPs from Organelle

A total of 26 MPs out of the 113 possess either chloroplast or mitochondrial origins (Table 3 and Table 4, Figure 5). They belong to the families of ADP/ATP carriers (AAC) and Mitochondrial Pyruvate Carriers (MPC) in mitochondria and chloroplast, but also to other families in chloroplasts.

#### 4.3.1. Mitochondrial MPs

AACs represent a large proportion of the MPs, with six TM domains expressed in *L. lactis* (Figure 3 and Figure 4). Firstly, two mitochondrial carriers from *S. cerevisiae*, CTP1 and AAC3, have been successfully expressed at a level of 5% and shown to be functionally active in *L. lactis* [11]. Subsequently, ten other carriers from *S. cerevisiae* have been successfully expressed, with levels ranging from 1 to 10%, and activities varying depending on the substrate and the protein studied [18]. The relatively high expression levels obtained for these proteins could most probably be linked to the presence of cardiolipin in the membrane of *L. lactis* (32%; [103]). Indeed, it could be demonstrated that the expression of these proteins is facilitated, and the presence of the appropriate lipids could help to drive the protein folding to the right conformation.

The human isoforms of the ATP/ADP translocators (AAC1, 2, and 3) displaying number of TM helices and size features similar to the mitochondrial carriers of *S. cerevisiae* were also studied. AAC1, expressed at 0.5–1% of TMP, was sensitive to the same inhibitors as its yeast orthologs [103]. Mutants of this MP were shown to be involved in childhood-onset mild skeletal myopathy [104]. Zhang and collaborators [105] tested and compared the efficiency of *L. lactis* versus yeast mitochondria in order to study the impact of the inhibitors of AACs on the different isoforms. Their studies revealed that *L. lactis* shows a higher specificity in the exchange assay than yeast, that it allows for the differentiation between direct and indirect inhibitors, and that it is more reproducible and can be prepared in large quantities.

Among the mitochondrial proteins, the MCPs are remarkable. Indeed, the isoforms of MCP1 and MCP2 from three different species, i.e., the yeast *Saccharomyces cerevisiae, Mus musculus,* and *Arabidopsis thaliana,* have been expressed under a functional state in their heterodimeric form in *L. lactis* [96,97]. The mouse isoforms were able to transport pyruvate across the membrane in intact recombinant bacteria [97]. This uptake was sensitive to the mitochondrial pyruvate carrier inhibitor UK5099 and to 2-deoxyglucose, which collapses the proton electrochemical gradient. Moreover, artificially increasing the membrane potential by lowering the pH in the buffer from 7.2 to 6.2 significantly increased pyruvate uptake. The co-expression of mMPC1 and mMPC2 in the membrane of *L. lactis* was sufficient to allow for the import of pyruvate, with properties similar to the mitochondrial pyruvate carrier [121].

#### 4.3.2. Chloroplast MPs

Expression in *L. lactis* using the NICE system proved to be efficient for the functional expression of several plant MPs involved in different chloroplast metabolic pathways, i.e., ceQORH, HMA6, and NTT1 proteins from *Arabidopsis thaliana*.

The peripheral ceQORH protein interacts with the chloroplast envelope through electrostatic interactions [122]. While this protein was produced in *E. coli* in inclusion bodies [122], it was expressed in *L. lactis* at almost 30% of TMP (Table 4; [50]), a surprisingly high expression level and similar to those obtained for homologous prokaryotic MPs (Table 2, Table 3 and Table 4). Functional characterization performed on purified proteins reconstituted in proteoliposomes revealed that ceQORH has NADPH-dependent dehydrogenase activity and requires a lipid environment. Moreover, when produced in *L. lactis*, ceQORH behaved as the natural chloroplast envelope protein and interacted with the bacterial membrane through electrostatic interactions [50].

Other chloroplast MPs such as the P1B-type ATPase family have also successfully been expressed, with levels from 0.7 to 3% of TMP (Table 4; [7,50]). These MPs (six–eight TM helices) translocate ions across plasma or organelle membranes at the expense of ATP consumption and are involved in the control of metal homeostasis within the cell [123]. Among the eight P1B-type ATPases encoded by the Arabidopsis genome, four have been successfully expressed in *L. lactis* [50]. Biochemical characterizations using phosphorylation assays were performed using *L. lactis* membranes expressing HMA6, and these assays allowed for the identification of this protein as a high-affinity Cu+ transporter of the chloroplast envelope [124].

The NTT1 protein is one of the AACs identified in the chloroplast; it imports ATP in exchange for ADP. This transporter has already been functionally characterized after expression in *S. cerevisiae* and *E. coli* [125,126]. Even though it was expressed at a very low level (0.2% of TMP), uptake assays of radioactive nucleotides could be performed on intact *L. lactis* cells and showed a time-dependent uptake of ATP, with a rate similar to the one measured in *E. coli* cells [50].

To conclude, *L. lactis* appears to be an appropriate expression system for the functional characterization of mitochondrial and Arabidopsis MPs, especially for chloroplast MPs. This can be explained by the fact that the *L. lactis* membrane contains cardiolipin and glycolipids [21], which are present in mitochondria [127] and the inner membrane of chloroplasts, respectively [128], in contrast to *E. coli* membranes [22], which have a different composition. The importance of the lipid composition of host cells in the overexpression of functional MPs has also already been underlined by other authors [3,23].

### 4.4. Membrane Proteins from Other Families

The first human MP produced in *L. lactis* was the KDEL receptor, Erd2. This protein with seven TM helices is involved in the retrieval of proteins of the endoplasmic reticulum (ER) at later stages of the secretory pathway. While expressed at a very low level, the protein could still bind its specific peptide and conserve the pH-dependent activities, as those in rat Golgi membranes [11].

Two MPs involved in human liver detoxification functions have been successfully expressed in *L. lactis*: the cytochrome-mono-oxygenase (CYP3A4) and the microsomal Glutathione S-Transferase 1 (MGST1). Interestingly, both proteins could be successfully expressed in *L. lactis* at higher levels than those previously obtained with classical expression systems (*E. coli*, *S. cerevisiae*) at 5 and 3% TMPs, respectively. This was also higher than results obtained for other eukaryotic membrane proteins expressed in *L. lactis* [51]. Expression of the MGST1 isoform from *Rattus norvegicus* in *L. lactis* was able to exhibit its GSH-transferase activity somewhat lower than values previously reported for rMGST1 from purified microsomes, or after heterologous expression in *E. coli*.

As discussed in the last two paragraphs, concerning the expression and functional characterization MPs in *L. lactis*, the number of MPs expressed in their functional state is increasing. Additional information has been obtained through the structural analysis of some of the proteins listed above.

## 5. Structures Resolved from MPs Expressed in *L. lactis*

Because of its numerous advantages in MP expression and functional characterization, *L. lactis* is now also a good alternative bacterial expression system for the structural determination of MPs of interest in *E. coli*. The first structure of a homologous MP expressed in *L. lactis* was obtained for OpuAC 10 years ago [129]. Then, the structure of ThiT was obtained, with both the wild-type and a selenomethionine-labeled protein. This crystal structure has been obtained at an expression level of 2% of TMPs [114,130]. One year after that, the same group resolved the structure of BioY, another *L. lactis* MP from the ECF family involved in biotin transport [59]. Altogether, almost 20 MP structures have been resolved in the last ten years after their expression in *L. lactis* using the NICE system, including their various conformations and bound to their substrates (Table 5).

Most of the 3D structures were obtained by X-ray diffraction; only the last four ones for OpuA were obtained by single-particle cryo-EM (cryogenic electron microscopy), a new emerging technique that allows for the acquisition of structures without crystallization, reducing the required sample amount and allowing the usage of a wide arsenal of hydrophobic environments, a large advantage when working with MPs [136].

This opens up the road to the elucidation of other MP structures in the future since the expression levels obtained for almost all the proteins is close to 1–2% and higher (Table 2, Table 3 and Table 4). Furthermore, the ability to label the MPs with SelenoMet resolves the diffraction data [137] and the availability of specific protocols developed for this purpose [138].

## 6. Conclusions

Over the last two decades, *Lactococcus lactis* emerged and proved to be an alternative and promising expression system to other bacterial systems. Numerous prokaryotic and eukaryotic MPs with diverse topologies, origins, and functions were successfully expressed in *L. lactis* using the tightly regulated NICE system and at a level, although lower than *E.coli*, that still allowed for functional and structural characterizations. Finally, twenty crystal structures of MPs after expression in *L. lactis* were resolved and have thus opened up the road to others in the future. This promising cell factory will enrich the knowledge on MPs in their functional and structural states, and bring about the development of further biotechnological and biotherapeutical applications in the near future.

## Figures and Tables

**Table 1:** Bacterial strains and plasmids commonly used in the NICE system for the overexpression of MPs. nisA, nisRK, genes of the nisin operon; RifR, StrpR, and CmR: resistance to rifampicine, streptomycine, and chloramphenicol, respectively.

		Characteristics	References
Strains			
*L. lactis*	NZ9700	Progeny of the conjugation between nisin producer strain NIZO B8 and MG1614 (RifR StrpR derivative of MG1363). Nisin producer strain for nisin-induced gene expression.	[11,29,39]
	NZ9800	Derivative of NZ9700 with deletion of 4 bp in the nisA gene. No nisin production but nisRK-transcribed. Host of NICE system.	[29,39]
	NZ9000	MG1363 strain with nisRK integrated into pepN gene. Most commonly used host for NICE system.	[29]
	NZ9100	MG1363 strain with nisR and nisK integrated into a neutral locus. Standard host strain of NICE.	Mobitec Molecular Biotechnology
	DML1	NZ9000 strain transformed with pNZ-X-GFP-EmrC and selected by increased concentration of erythromycin.	[35]
Plasmids.			
pNZ8048		*Nco*I site used for translational fusions, CmR.	[29]
pNZ8148		pNZ8048 with deletion of 60 bp DNA from *B. subtilis*, CmR.	[20]
pNZ8149		pNZ8048 with *Nco*I site for translational fusions; lacF for food grade selection for growth on lactose.	Mobitec Molecular Biotechnology
pNZ8150		pNZ8148 with *Sca*I site used for translational fusions, CmR.	[20]
pNZ8151		pNZ8148 with *Sca*I site used for translational fusions, lacF.	Mobitec Molecular Biotechnology
pNZ8152		pNZ8148 with *Sca*I site used for translational fusions, alr gene for food grade selection.	Mobitec Molecular Biotechnology

**Table 2:** List of homologous prokaryotic MPs.

Protein	Function	Size (kDa) ^a^	TM Helices ^b^	Expression Level ^c^	References
ArcD1	arginine/ornithine antiporter	52.6	13	-	[57]
ArcD2	arginine/ornithine antiporter	54	13	-	[57,58]
BcaP	branched-chain amino acid permease	50	12	20%	[38]
BioY	biotin transporter	20.5	5	5%	[59]
ChoS	glycine betaine ABC transporter permease	55.1	5	2%	[60]
CitP	citrate sodium symporter	48.6	13	1–2%	[61]
CmbT	MFS transporter	50	12	<1%	[62]
DtpT	di-/tripeptide transporter	54.8	12	10%	[11]
GlnP	ABC transporter	78.5	3	<1%	[60,63]
GlnQ	glutamine transport ATP- binding	27	8	2–5%	
LmrA	ABC efflux pump	65	6	30%	[64]
LmrCD	ABC transporter	63 + 73.7	6 + 6	5–10%	[65]
LmrP	MFS efflux pump	45	12	5%	[66,67,68]
MleP	MFS transporter	46.7	11	1–2%	[11]
MscL	large-conductance mechanosensitive channel	13.8	2	5–10%	[69]
OppB	ABC transporter with OpuC,D,F	35.1	6	<1%	[11]
OppC	ABC transporter with OpuB,D,F	32.3	6	<1%	
OpuABC	ABC transporter with OpuAA	63	8	10%	[11,70]
RibU	riboflavin transporter	23	6	5%	[71]
SerP1	serine permease	51.3	12	-	[72]
SerP2	DL-alanine permease	51.5	12	-	
ThiT	thiamine transporter	20	6	2%	[45]

^a^ Protein sizes are given in kDa and for full proteins. ^b^ The number of TM helices listed here has either already been demonstrated or predicted with software (such as TNHMM or psipred) with the FASTA sequence published on Uniprot. p for peripheral proteins. ^c^ The expression levels are given as a percentage of the recombinant protein compared to the total membrane proteins (TMP).

**Table 3:** List of heterologous prokaryotic MPs.

Protein	Function	Size (kDa) ^a^	TM Helices ^b^	Organism ^c^	Expression Level ^d^	References
abcA	ABC transporter	70	6	*B. breve*	1%	[73]
abcB	ABC transporter	66	6		5–10%	
LanR1	lantibiotic response regulator	24	6	*B. longum*	-	[74]
LanI	ABC transporter	32.76	-		-	
LanT	lantibiotic transporter	80.1	6		-	
BmrA	ABC transporter	65.3	6		5–10%	[75]
tlyC1	hemolysin-like protein	11.2	2		-	[76]
Omp16	Peptidoglycan-associated lipoprotein	18.2	p	*B. melitensis*	-	[77]
DctA	C4-dicarboxylate transport	45.4	8	*B. subtilis*	0.5–1%	[44]
CA_C2849	proline/glycine betaine ABC-type transport system, permease	57.6	6	*C. acetobutylicum*	2%	[60]
MsbA	lipid A export ATP-binding/ permease	64.5	6	*E. coli*	20–30%	[78]
EfrA	ABC transporter	56.3	4	*E. faecalis*	-	[79]
EfrB	ABC transporter	60.54	3		-	
Jhp0757	putative osmoprotection binding protein	62.6	6	*H. pylori*	1%	[60]
HpaA	neuraminyllactose-binding hemagglutinin	29.1	p		25–30%	[80]
HorA	Multidrug transporter	64.2	5	*L. brevis*	30%	[81]
ArcD	arginine/ornithine exchangers	51.9	13		-	[82]
Lin0840	ABC transporter	53.2	6	*L. innocua*	<1%	[60]
Lin1461	binding-protein-dependent transport system permease	55.7	6		2%	
Lin2352	ABC transporter	53.4	6		1%	
Lmo1422	binding-protein-dependent transport system permease	55.7	6	*L. monocytogenes*	1%	[60]
Lmo2250	ABC transporter	53.1	6		2%	
cwaA	cell wall-anchored adhesion-associated protein	93.7	2	*L. plantarum*	-	[83]
OppA	oligopeptide-binding protein	59.7	p	*L. salivarius*	-	[84]
XylP	xylose-proton symporter	52.7	12	*Lb. pentosus*	20%	[11]
Rv1410	MFS transporter	54.7	14	*M. smegmatis*	-	[85]
CYP201A2	cytochrome-mono-oxygenase	49.7	p	*R. palustris*	1.5%	[7]
TlcA,B,C	ATP/ADP translocator	56.8	12	*R. prowazekii*	5–10%	[11]
NapC	cytochrome-electron transfer	25.6	1	*R. sphaeroides*	0.5%	[7]
BspA	Gram+ anchoring domain containing protein	101	1	*S. agalactiae*	-	[86]
SAR1949	putative extracellular glutamine-binding protein	53.1	4	*S. aureus*	1%	[60]
Sav1866	multidrug export ATP- binding/permease	64.8	6		20–25%	[87]
Cnm	collagen and laminin-binding glycoprotein	58	1	*S. mutans*	-	[88]
PspC	Choline-binding protein	85.24	1	*S. pneumoniae*	-	[89]
MreC	peptidoglycan synthesis	32	1		1%	[7]
ProWX	ABC transporter permease- choline transporter	55.5	6		2–3%	[60]
SP_0453	ABC transporter, AA-binding protein/permease protein	57.4	6		<1%	
SP_1241	ABC transporter, AA-binding protein/permease protein	78.4	3		<1%	
LacS	MFS transporter	56.6	12	*S. thermophilus*	1–2%	[11]
SfbA/FbaA	streptococcal fibronectin-binding protein A	37.8	1	*Streptococcus*	-	[90]
SfbI	Fibronecting-binding protein	67.3	1		-	
TM287/288	ABC transporter	60 + 60	6 + 6	*T. maritima*	0.5–1%	[91]

^a^ Protein sizes are given in kDa and for full proteins. ^b^ The number of TM helices listed here has either already been demonstrated or predicted with software (such as TNHMM or psipred) with the FASTA sequence published on Uniprot. p for peripheral proteins. ^c^ *B. breve* (*Bifidobacterium breve*); *B. longum* (*Bifidobacterium longum*); *B. melitensis* (*Brucella melitensis*); *B. subtilis* (*Bacillus subtilis*); *C. acetobutylicum* (*Clostridium acetobutylicum*); *E. coli* (*Escherichia coli*); *E. faecalis* (*Enterococcus faecalis*); *H. pylori* (*Helicobacter pylori*); *L. brevis* (*Lactobacillus brevis*); *L. innocua* (*Listeria innocua*); *L. monocytogenes* (*Listeria monocytogenes*); *L. plantarum* (*Lactobacillus plantarum*); *Lb; pentosus* (*Lactobacillus pentosus*); *L. salivarius* (*Lactobacillus salivarius*); *M. smegmatis* (*Mycobacterium smegmatis*); *R. palustris* (*Rhodopseudomonas palustris*); *R. prowazekii* (*Rickettsia prowazekii*); *R. sphaeroides* (*Rhodobacter sphaeroides*); *S. agalactiae* (*Streptococcus agalactiae*); *S. aureus* (*Staphylococcus aureus*); *S. mutans* (*Streptococcus mutans*); *S. pneumoniae* (*Streptococcus pneumonia*); *S. thermophilus* (*Streptococcus thermophilus*); *T. maritime* (*Thermotoga maritima*). ^d^ The expression levels are given as a percentage of the recombinant protein compared to the total membrane proteins (TMP).

**Table 4:** List of eukaryotic MPs.

Protein	Function	Size (kDa) ^a^	TM Helices ^b^	Organism ^c^	Expression Level ^d^	References
ATM1	mitochondrial iron-sulfur cluster transporter	77.5	6	*S. cerevisiae*	-	[92]
GDT1	cation exchanger (homologous to TMEM)	30.3	7	*S. cerevisiae*	-	[93]
CTP1	tricarboxylate transport protein	32.9	6	*S. cerevisiae*	5%	[18]
SAM5	mitochondrial S-adenosyl methionine carrier	30.9	4	*S. cerevisiae*	<1%	
Mdl1	mitochondrial ATP-dependent permease	76	5	*S. cerevisiae*	<0.1%	[94]
MIR1	mitochondrial phosphate carrier protein	32.8	6	*S. cerevisiae*	<1%	[18]
DIC1	mitochondrial dicarboxylate transporter	33	6	*S. cerevisiae*	10%	
GGC1	mitochondrial GTP/GDP carrier protein	33.2	6	*S. cerevisiae*	4%	
PIC2	mitochondrial phosphate carrier protein 2	33.5	6	*S. cerevisiae*	1–2%	[95]
AAC3	mitochondrial ADP/ATP carrier protein 3	33.7	6	*S. cerevisiae*	5%	[11]
ODC2	mitochondrial 2-oxodicarboxylate carrier 2	34	6	*S. cerevisiae*	10%	[18]
AAC1	mitochondrial ADP/ATP carrier protein 1	34.1	6	*S. cerevisiae*	<1%	
ODC1	mitochondrial 2-oxodicarboxylate carrier 1	34.2	6	*S. cerevisiae*	8%	
AAC2	mitochondrial ADP/ATP carrier protein 2	34.4	6	*S. cerevisiae*	<1%	
MPC1/2	mitochondrial pyruvate carrier	15 + 14.5	2 + 3	*S. cerevisiae*	-	[96]
MPC1/2	mitochondrial pyruvate carrier	12.3 + 14.3	2 + 2	*M. musculus*	<1%	[96,97]
MPC1/2	mitochondrial pyruvate carrier	12.4 + 12.2	2 + 3	*A. thaliana*	-	[96]
ceQORH	quinone oxidoreductase-electron transfer	33.1	p	*A. thaliana*	30%	[50]
LPR1	multi-copper oxidase	60.5	p	*A. thaliana*	<0.1%	[7]
PHF	phosphate transport regulation	42.4	1	*A. thaliana*	1.5%	
AtHMA1	heavy metal transporter	80.1	6	*A. thaliana*	3%	[50]
AtHMA3	heavy metal transporter	81.4	8	*A. thaliana*	1%	
AtHMA6	heavy metal transporter	100	8	*A. thaliana*	3%	
AtHMA4	heavy metal transporter	126.7	8	*A. thaliana*	0.75%	[7]
NTT1	chloroplast ADP/ATP transporter	57.5	12	*A. thaliana*	0.2%	[50]
NRT1 (NPF2.3)	nitrate excretion transporter	61	12	*A. thaliana*	-	[98]
ATM3 (ABCB25)	mitochondrial ABC transporter	80	7	*A. thaliana*	-	[92]
AAC hyd	hydrogenosomal carrier	33.9	6	*N. patriciarum*	<1%	[11]
SUT1	sucrose transporter	54.8	12	*S. tuberosum*	1–2%	[99]
L276	mitochondrial carrier-like	27.3	6	*A. polyphaga*	5%	[100]
Bcl-Xl	apoptosis regulation	24.7	1	*H. sapiens*	1%	[7]
CYP3A4	cytochrome-mono-oxygenase	57.4	1	*H. sapiens*	5%	[51]
MGST1	microsomal glutathione S-transferase 1	17.6	4	*H. sapiens*	3%	
ABCG2	breast cancer resistance protein	72	6	*H. sapiens*	0.5–1%	[101]
Erd2	KDEL receptor	24.4	7	*H. sapiens*	<0.1%	[11]
CXCR4	chemokine receptor type 4	37.9	7	*H. sapiens*	<0.1%	[7]
CCR5	chemokine receptor type 5	38.7	7	*H. sapiens*	<0.1%	
PS1Δ9	human alpha secretase component	55	9	*H. sapiens*	0.1–0.2%	[100]
CFTR	cystic fibrosis transmembrane conductance regulator	168	12	*H. sapiens*	<0.1%	[46]
TMEM165	cation transporter	34.9	6	*H. sapiens*	-	[102]
AAC1	mitochondrial ADP/ATP carrier protein 1	34	6	*H. sapiens*	0.5–1%	[103,104,105]
ANT2(AAC2)	mitochondrial ADP/ATP carrier protein 2	32.8	6	*H. sapiens*	-	[104]
ANT3(AAC3)	mitochondrial ADP/ATP carrier protein 3	32.8	6	*H. sapiens*	-	[104]
SLC25A3	mitochondrial pyruvate carrier (homologous to PIC)	40.1	6	*H. sapiens*	-	[106]

^a^ Protein sizes are given in kDa and for full proteins, i.e., including the signal peptide for mitochondrial and chloroplastic MPs (truncated for heterologous expression); ^b^ The number of TM helices listed here has either already been demonstrated or predicted with software (such as TNHMM or psipred) with the FASTA sequence published on Uniprot. p for peripheral proteins. ^c^ *A. polyphaga* (*Acanthamoeba polyphaga*); *A. thaliana* (*Arabidopsis thaliana*); *H. sapiens* (*Homo sapiens*); *M. musculus* (*Mus musculus*); *N. patriciarum* (*Neocallimastix patriciarum*); *S. cerevisiae* (*Saccharomyces cerevisiae*); *S. tuberosum* (*Solanum tuberosum*). ^d^ The expression levels are given as a percentage of the recombinant protein compared to the total membrane proteins (TMP).

**Table 5:** Structures obtained after expression in *L. lactis*.

Protein	Organism	Code	Structure	References
OpuA	*L. lactis*	7AHH	OpuA inhibited inward-facing, SBD docked	[131]
		7AHC	OpuA apo inward-facing	
		7AHE	OpuA inhibited inward-facing	
		7AHD	OpuA (E190Q) occluded	
PrgL	*E. faecalis*	7AED	VirB8 domain of PrgL from *Enterococcus faecalis* Pcf10	[132]
MhsT	*A. halodurans*	6YU2	Crystal structure of MhsT in complex with L-isoleucine	[133]
		6YU3	Crystal structure of MhsT in complex with L-phenylalanine	
		6YU4	Crystal structure of MhsT in complex with L-4F-phenylalanine	
		6YU5	Crystal structure of MhsT in complex with L-valine	
		6YU6	Crystal structure of MhsT in complex with L-leucine	
		6YU7	Crystal structure of MhsT in complex with L-tyrosine	
GlnPQ	*L. lactis*	6FXG	Crystal structure of substrate binding domain 1 (SBD1) OF ABC transporter GLNPQ in complex with Asparagine	[134]
ECF	*L. delbrueckii* subsp. *Bulgaricus*	5D0Y	Substrate bound S-component of folate ECF transporter	[115]
ATP-Mg/Pi carrier (APC)		4ZCU	Structure of calcium-bound regulatory domain of the human ATP-Mg/Pi carrier in the P2 form	[135]
		4ZCV	Structure of calcium-bound regulatory domain of the human ATP-Mg/Pi carrier in the P212121 form	
ThiT	*L. lactis* subsp. *cremoris MG1363*	4POP	ThiT with LMG139 bound	[130]
		4POV	ThiT with LMG135 bound	
ECF	*L. lactis* subsp. *cremoris MG1363*	4DVE	Crystal structure at 2.1 A of the S-component for biotin from an ECF-type ABC transporter	[59]
OpuAC	*L. lactis*	3L6G	Crystal structure of lactococcal OpuAC in its open conformation	[129]
		3L6H	Crystal structure of lactococcal OpuAC in its closed-liganded conformation complexed with glycine betaine	

## Data Availability

Not applicable.

## References

[1] Wallin E., von Heijne G. (1998). Genome-wide analysis of integral membrane proteins from eubacterial, archaean, and eukaryotic organisms. Protein Sci..

[2] Lundstrom K. (2007). Structural genomics and drug discovery. J. Cell Mol. Med..

[3] Junge F., Schneider B., Reckel S., Schwarz D., Dötsch V., Bernhard F. (2008). Large-scale production of functional membrane proteins. Cell Mol. Life Sci..

[4] Kesidis A., Depping P., Lodé A., Vaitsopoulou A., Bill R.M., Goddard A.D., Rothnie A.J. (2020). Expression of eukaryotic membrane proteins in eukaryotic and prokaryotic hosts. Methods.

[5] Lacapere J.J., Pebay-Peyroula E., Neumann J.M., Etchebest C. (2007). Determining membrane protein structures: Still a challenge. Trends Biochem. Sci..

[6] Fogeron M.L., Lecoq L., Cole L., Harbers M., Böckmann A. (2021). Easy Synthesis of Complex Biomolecular Assemblies: Wheat Germ Cell-Free Protein Expression in Structural Biology. Front. Mol. Biosci..

[7] Bernaudat F., Frelet-Barrand A., Pochon N., Dementin S., Hivin P., Boutigny S., Rioux J.B., Salvi D., Seigneurin-Berny D., Richaud P. (2011). Heterologous expression of membrane proteins: Choosing the appropriate host. PLoS ONE.

[8] Gordon E., Horsefield R., Swarts H.G., de Pont J.J., Neutze R., Snijder A. (2008). Effective high-throughput overproduction of membrane proteins in Escherichia coli. Protein Expr. Purif..

[9] Kaur J., Kumar A., Kaur J. (2018). Strategies for optimization of heterologous protein expression in *E. coli*: Roadblocks and reinforcements. Int. J. Biol. Macromol..

[10] Schlegel S., Klepsch M., Gialama D., Wickström D., Slotboom D.J., de Gier J.W. (2010). Revolutionizing membrane protein overexpression in bacteria. Microb. Biotechnol..

[11] Kunji E.R.S., Slotboom D.J., Poolman B. (2003). Lactococcus lactis as host for overproduction of functional membrane proteins. Biochim. Biophys. Acta.

[12] Bakari S., André F., Seigneurin-Berny D., Delaforge M., Rolland N., Frelet-Barrand A., Mus-Vuteau I. (2014). Lactococcus lactis, recent developments in functional expression of membrane proteins. Membrane Proteins Production for Structural Analysis.

[13] Gasson M.J., de Vos W.M. (1994). Genetics and Biotechnology of Lactic Acid Bacteria.

[14] Mierau I., Olieman K., Mond J., Smid E.J. (2005). Optimization of the Lactococcus lactis nisin-controlled gene expression system NICE for industrial applications. Microb. Cell Fact..

[15] Morello E., Bermúdez-Humarán L.G., Llull D., Solé V., Miraglio N., Langella P., Poquet I. (2008). Lactococcus lactis, an efficient cell factory for recombinant protein production and secretion. J. Mol. Microbiol. Biotechnol..

[16] Song A.A., In L.L.A., Lim S.H.E., Rahim R.A. (2017). A review on Lactococcus lactis: From food to factory. Microb. Cell Fact..

[17] Kunji E.R.S., Chan K.W., Slotboom D.J., Floyd S., O’Connor R., Monné M. (2005). Eukaryotic membrane protein overproduction in Lactococcus lactis. Curr. Opin. Biotechnol..

[18] Monné M., Chan K.W., Slotboom D.J., Kunji E.R.S. (2005). Functional expression of eukaryotic membrane proteins in Lactococcus lactis. Protein Sci..

[19] van Gijtenbeek L.A., Robinson A., van Oijen A.M., Poolman B., Kok J. (2016). On the Spatial Organization of mRNA, Plasmids, and Ribosomes in a Bacterial Host Overexpressing Membrane Proteins. PLoS Genet..

[20] Mierau I., Kleerebezem M. (2005). 10 years of the nisin-controlled gene expression system (NICE) in Lactococcus lactis. Appl. Microbiol. Biotechnol..

[21] Oliveira A.P., Nielsen J., Förster J. (2005). Modelling Lactococcus lactis using a genome-scale flux model. BMC Microbiol..

[22] Ingram L.O. (1977). Changes in lipid composition of Escherichia coli resulting from growth with organic solvents and with food additives. Appl. Environ. Microbiol..

[23] Opekarova M., Tanner W. (2003). Specific lipid requirements of membrane proteins—A putative bottleneck in heterologous expression. Biochim. Biophys. Acta.

[24] Surade S., Klein M., Stolt-Bergner P.C., Muenke C., Roy A., Michel H. (2006). Comparative analysis and “expression space” coverage of the production of prokaryotic membrane proteins for structural genomics. Protein Sci..

[25] Pontes D.S., de Azevedo M.S., Chatel J.M., Langella P., Azevedo V., Miyoshi A. (2011). Lactococcus lactis as a live vector: Heterologous protein production and DNA delivery systems. Protein Expr. Purif..

[26] Lubelski J., Rink R., Khusainov R., Moll G.N., Kuipers O.P. (2008). Biosynthesis, immunity, regulation, mode of action and engineering of the model lantibiotic nisin. Cell Mol. Life Sci..

[27] Delves-Broughton J., Blackburn P., Evans R.J., Hugenholtz J. (1996). Applications of the bacteriocin, nisin. Antonie Van Leeuwenhoek.

[28] Gasson M.J. (1983). Genetic transfer systems in lactic acid bacteria. Antonie Van Leeuwenhoek.

[29] Kuipers O.P., de Ruyter P.G.G.A., Kleerebezem M., de Vos W.M. (1998). Quorum sensing-controlled gene expression in lactic acid bacteria. J. Biotechnol..

[30] Hasper H.E., de Kruijff B., Breukink E. (2004). Assembly and stability of nisin-lipid II pores. Biochemistry.

[31] de Ruyter P.G., Kuipers O.P., Beerthuyzen M.M., Alen-Boerrigter I., de Vos W.M. (1996). Functional analysis of promoters in the nisin gene cluster of Lactococcus lactis. J. Bacteriol..

[32] de Ruyter P.G., Kuipers O.P., de Vos W.M. (1996). Controlled gene expression systems for Lactococcus lactis with the food-grade inducer nisin. Appl. Environ. Microbiol..

[33] Zhou X.X., Li W.F., Ma G.X., Pan Y.J. (2006). The nisin-controlled gene expression system: Construction, application and improvements. Biotechnol. Adv..

[34] Mu D., Montalbán-López M., Masudaa Y., Kuipers O.P. (2013). Zirex: A Novel Zinc-Regulated Expression System for Lactococcus lactis. Appl. Environ. Microbiol..

[35] Linares D.M., Geertsma E.R., Poolman B. (2010). Evolved Lactococcus lactis strains for enhanced expression of recombinant membrane proteins. J. Mol. Biol..

[36] Poquet I., Saint V., Seznec E., Simoes N., Bolotin A., Gruss A. (2000). HtrA is the unique surface housekeeping protease in Lactococcus lactis and is required for natural protein processing. Mol. Microbiol..

[37] Noreen N., Hooi W.Y., Baradaran A., Rosfarizan M., Sieo C.C., Rosli M.I., Yusoff K., Raha A.R. (2011). Lactococcus lactis M4, a potential host for the expression of heterologous proteins. Microb. Cell Fact..

[38] Pinto J.P., Kuipers O.P., Marreddy R.K., Poolman B., Kok J. (2011). Efficient overproduction of membrane proteins in Lactococcus lactis requires the cell envelope stress sensor/regulator couple CesSR. PLoS ONE.

[39] Kuipers O.P., Beerthuyzen M.M., Siezen R.J., de Vos W.M. (1993). Characterization of the nisin gene cluster nisABTCIPR of Lactococcus lactis. Requirement of expression of the nisA and nisI genes for development of immunity. Eur. J. Biochem..

[40] de Vos W.D. (1987). Gene cloning and expression in lactic streptococci. FEMS Microbiol. Lett..

[41] Kok J., van der Vossen J.M., Venema G. (1984). Construction of plasmid cloning vectors for lactic streptococci which also replicate in Bacillus subtilis and Escherichia coli. Appl. Environ. Microbiol..

[42] de Vos W.M., Simons G.F.M., Gasson M.J., de Vos W.M. (1994). Gene cloning and expression systems in Lactococci. Genetics and Biotechnology of Lactic Acid Bacteria.

[43] Geertsma E.R., Poolman B. (2007). High-throughput cloning and expression in recalcitrant bacteria. Nat. Methods.

[44] Groeneveld M., Weme R.G., Duurkens R.H., Slotboom D.J. (2010). Biochemical characterization of the C4-dicarboxylate transporter DctA from Bacillus subtilis. J. Bacteriol..

[45] Erkens G.B., Slotboom D.J. (2010). Biochemical characterization of ThiT from Lactococcus lactis: A thiamin transporter with picomolar substrate binding affinity. Biochemistry.

[46] Steen A., Wiederhold E., Gandhi T., Breitling R., Slotboom D.J. (2011). Physiological adaptation of the bacterium Lactococcus lactis in response to the production of human CFTR. Mol. Cell Proteom..

[47] Hartley J.L., Temple G.F., Brasch M.A. (2000). DNA cloning using in vitro site-specific recombination. Genome Res..

[48] Eshaghi S., Hedrén M., Nasser M.I., Hammarberg T., Thornell A., Nordlund P. (2005). An efficient strategy for high-throughput expression screening of recombinant integral membrane proteins. Protein Sci..

[49] Yashiroda Y., Matsuyama A., Yoshida M. (2008). New insights into chemical biology from ORFeome libraries. Curr. Opin. Chem. Biol..

[50] Frelet-Barrand A., Boutigny S., Moyet L., Deniaud A., Seigneurin-Berny D., Salvi D., Bernaudat F., Richaud P., Pebay-Peyroula E., Joyard J. (2010). Lactococcus lactis, an alternative system for functional expression of peripheral and intrinsic Arabidopsis membrane proteins. PLoS ONE.

[51] Bakari S., Lembrouk M., André F., Orlowski S., Delaforge M., Frelet-Barrand A. (2016). Expression in Lactococcus lactis of two human membrane proteins involved in liver detoxification, cytochrome P450 3A4 and microsomal glutathione S-transferase MGST1. Mol. Biotechnol..

[52] Douillard F.P., Mahony J., Campanacci V., Cambillau C., van Sinderen D. (2011). Construction of two Lactococcus lactis expression vectors combining the Gateway and the NIsin Controlled Expression systems. Plasmid.

[53] Murphy J., Klumpp J., Mahony J., O’Connell-Motherway M., Nauta A., van Sinderen D. (2014). Methyltransferases acquired by lactococcal 936-type phage provide protection against restriction endonuclease activity. BMC Genom..

[54] Berlec A., Štrukelj B. (2012). Generating a custom TA-cloning expression plasmid for Lactococcus lactis. Biotechniques.

[55] Berlec A., Škrlec K., Kocjan J., Olenic M., Štrukelj B. (2018). Single plasmid systems for inducible dual protein expression and for CRISPR-Cas9/CRISPRi gene regulation in lactic acid bacterium Lactococcus lactis. Sci. Rep..

[56] Plavec T.V., Mitrović A., Perišić Nanut M., Štrukelj B., Kos J., Berlec A. (2021). Targeting of fluorescent Lactococcus lactis to colorectal cancer cells through surface display of tumour-antigen binding proteins. Microb. Biotechnol..

[57] Noens E.E., Lolkema J.S. (2015). Physiology and substrate specificity of two closely related amino acid transporters, SerP1 and SerP2, of Lactococcus lactis. J. Bacteriol..

[58] Pols T., Singh S., Deelman-Driessen C., Gaastra B.F., Poolman B. (2021). Enzymology of the pathway for ATP production by arginine breakdown. FEBS J..

[59] Berntsson R.P., ter Beek J., Majsnerowska M., Duurkens R.H., Puri P., Poolman B., Slotboom D.J. (2012). Structural divergence of paralogous S components from ECF-type ABC transporters. Proc. Natl. Acad. Sci. USA.

[60] Marreddy R.K.R., Geertsma E.R., Poolman B., Brnjas-Kraljević J., Pifat-Mrzljak G. (2011). Recombinant Membrane Protein Production: Past, Present and Future. Supramolecular Structure and Function.

[61] Pudlik A.M., Lolkema J.S. (2012). Rerouting citrate metabolism in Lactococcus lactis to citrate-driven transamination. Appl. Environ. Microbiol..

[62] Filipic B., Golic N., Jovcic B., Tolinacki M., Bay D.C., Turner R.J., Antic-Stankovic J., Kojic M., Topisirovic L. (2013). The cmbT gene encodes a novel major facilitator multidrug resistance transporter in Lactococcus lactis. Res. Microbiol..

[63] Fulyani F., Schuurman-Wolters G.K., Slotboom D.J., Poolman B. (2016). Relative Rates of Amino Acid Import via the ABC Transporter GlnPQ Determine the Growth Performance of Lactococcus lactis. J. Bacteriol..

[64] Venter H., Shilling R.A., Velamakanni S., Balakrishnan L., Van Veen H.W. (2003). An ABC transporter with a secondary-active multidrug translocator domain. Nature.

[65] Lubelski J., de Jong A., van Merkerk R., Agustiandari H., Kuipers O.P., Kok J., Driessen A.J. (2006). LmrCD is a major multidrug resistance transporter in Lactococcus lactis. Mol. Microbiol..

[66] Schaedler T.A., Tong Z., van Veen H.W. (2012). The multidrug transporter LmrP protein mediates selective calcium efflux. J. Biol. Chem..

[67] Debruycker V., Hutchin A., Masureel M., Ficici E., Martens C., Legrand P., Stein R.A., Mchaourab H.S., Faraldo-Gómez J.D., Remaut H. (2020). An embedded lipid in the multidrug transporter LmrP suggests a mechanism for polyspecificity. Nat. Struct. Mol. Biol..

[68] Swain B.M., Guo D., Singh H., Rawlins P.B., McAlister M., van Veen H.W. (2020). Complexities of a protonatable substrate in measurements of Hoechst 33342 transport by multidrug transporter LmrP. Sci. Rep..

[69] Folgering J.H., Moe P.C., Schuurman-Wolters G.K., Blount P., Poolman B. (2005). Lactococcus lactis uses MscL as its principal mechanosensitive channel. J. Biol. Chem..

[70] Tassis K., Vietrov R., de Koning M., de Boer M., Gouridis G., Cordes T. (2021). Single-molecule studies of conformational states and dynamics in the ABC importer OpuA. FEBS Lett..

[71] Duurkens R.H., Tol M.B., Geertsma E.R., Permentier H.P., Slotboom D.J. (2007). Flavin binding to the high affinity riboflavin transporter RibU. J. Biol. Chem..

[72] Noens E.E., Kaczmarek M.B., Żygo M., Lolkema J.S. (2015). ArcD1 and ArcD2 Arginine/Ornithine Exchangers Encoded in the Arginine Deiminase Pathway Gene Cluster of Lactococcus lactis. J. Bacteriol..

[73] Margolles A., Flórez A.B., Moreno J.A., van Sinderen D., de los Reyes-Gavilán C.G. (2006). Two membrane proteins from Bifidobacterium breve UCC2003 constitute an ABC-type multidrug transporter. Microbiology.

[74] Yu L., Liu X., O’Sullivan D.J. (2018). Use of Lactococcus lactis as a production system for peptides and enzymes encoded by a Lantibiotic gene cluster from Bifidobacterium longum. Microbiology.

[75] Xu Q., Zhai Z., An H., Yang Y., Yin J., Wang G., Ren F., Hao Y. (2019). The MarR Family Regulator BmrR Is Involved in Bile Tolerance of Bifidobacterium longum BBMN68 via Controlling the Expression of an ABC Transporter. Appl. Environ. Microbiol..

[76] Liu Y., An H., Zhang J., Zhou H., Ren F., Hao Y. (2014). Functional role of tlyC1 encoding a hemolysin-like protein from Bifidobacterium longum BBMN68 in bile tolerance. FEMS Microbiol. Lett..

[77] Rezaei M., Rabbani Khorasgani M., Zarkesh Esfahani S.H., Emamzadeh R., Abtahi H. (2020). Production of Brucella melitensis Omp16 protein fused to the human interleukin 2 in Lactococcus lactis MG1363 toward developing a Lactococcus-based vaccine against brucellosis. Can. J. Microbiol..

[78] Woebking B., Reuter G., Shilling R.A., Velamakanni S., Shahi S., Venter H., Balakrishnan L., van Veen H.W. (2005). Drug-lipid A interactions on the Escherichia coli ABC transporter MsbA. J. Bacteriol..

[79] Hürlimann L.M., Corradi V., Hohl M., Bloemberg G.V., Tieleman D.P., Seeger M.A. (2016). The Heterodimeric ABC Transporter EfrCD Mediates Multidrug Efflux in Enterococcus faecalis. Antimicrob. Agents Chemother..

[80] Zhang R., Wang C., Cheng W., Duan G., Shi Q., Chen S., Fan Q. (2018). Delivery of Helicobacter pylori HpaA to gastrointestinal mucosal immune sites using Lactococcus lactis and its immune efficacy in mice. Biotechnol. Lett..

[81] Sakamoto K., Margolles A., van Veen H.W., Konings W.N. (2001). Hop resistance in the beer spoilage bacterium Lactobacillus brevis is mediated by the ATP-binding cassette multidrug transporter HorA. J. Bacteriol..

[82] Majsnerowska M., Hänelt I., Wunnicke D., Schäfer L.V., Steinhoff H.J., Slotboom D.J. (2013). Substrate-induced conformational changes in the S-component ThiT from an energy coupling factor transporter. Structure.

[83] Zhang B., Zuo F., Yu R., Zeng Z., Ma H., Chen S. (2015). Comparative genome-based identification of a cell wall-anchored protein from Lactobacillus plantarum increases adhesion of Lactococcus lactis to human epithelial cells. Sci. Rep..

[84] Martín C., Escobedo S., Pérez-Martínez G., Coll-Marqués J.M., Martín R., Suárez J.E., Quirós L.M. (2019). Two alkaline motifs in the Lactobacillus salivarius Lv72 OppA surface are important to its adhesin function. Benef. Microbes.

[85] Hohl M., Remm S., Eskandarian H.A., Dal Molin M., Arnold F.M., Hürlimann L.M., Krügel A., Fantner G.E., Sander P., Seeger M.A. (2019). Increased drug permeability of a stiffened mycobacterial outer membrane in cells lacking MFS transporter Rv1410 and lipoprotein LprG. Mol. Microbiol..

[86] Rego S., Heal T.J., Pidwill G.R., Till M., Robson A., Lamont R.J., Sessions R.B., Jenkinson H.F., Race P.R., Nobbs A.H. (2016). Structural and Functional Analysis of Cell Wall-anchored Polypeptide Adhesin BspA in Streptococcus agalactiae. J. Biol. Chem..

[87] Velamakanni S., Yao Y., Gutmann D.A., van Veen H.W. (2008). Multidrug transport by the ABC transporter Sav1866 from Staphylococcus aureus. Biochemistry.

[88] Freires I.A., Avilés-Reyes A., Kitten T., Simpson-Haidaris P.J., Swartz M., Knight P.A., Rosalen P.L., Lemos J.A., Abranches J. (2017). Heterologous expression of Streptococcus mutans Cnm in Lactococcus lactis promotes intracellular invasion, adhesion to human cardiac tissues and virulence. Virulence.

[89] Asmat T.M., Klingbeil K., Jensch I., Burchhardt G., Hammerschmidt S. (2012). Heterologous expression of pneumococcal virulence factor PspC on the surface of Lactococcus lactis confers adhesive properties. Microbiology.

[90] Mu R., Kim B.J., Paco C., Del Rosario Y., Courtney H.S., Doran K.S. (2014). Identification of a group B streptococcal fibronectin binding protein, SfbA, that contributes to invasion of brain endothelium and development of meningitis. Infect. Immun..

[91] Hohl M., Briand C., Grütter M.G., Seeger M.A. (2012). Crystal structure of a heterodimeric ABC transporter in its inward-facing conformation. Nat. Struct. Mol. Biol..

[92] Schaedler T.A., Thornton J.D., Kruse I., Schwarzländer M., Meyer A.J., van Veen H.W., Balk J. (2014). A conserved mitochondrial ATP-binding cassette transporter exports glutathione polysulfide for cytosolic metal cofactor assembly. J. Biol. Chem..

[93] Colinet A.S., Sengottaiyan P., Deschamps A., Colsoul M.L., Thines L., Demaegd D., Duchêne M.C., Foulquier F., Hols P., Morsomme P. (2016). Yeast Gdt1 is a Golgi-localized calcium transporter required for stress-induced calcium signaling and protein glycosylation. Sci. Rep..

[94] Hofacker M., Gompf S., Zutz A., Presenti C., Haase W., van der Does C., Model K., Tampé R. (2007). Structural and functional fingerprint of the mitochondrial ATP-binding cassette transporter Mdl1 from Saccharomyces cerevisiae. J. Biol. Chem..

[95] Vest K.E., Leary S.C., Winge D.R., Cobine P.A. (2013). Copper Import into the Mitochondrial Matrix in Saccharomyces cerevisiae is Mediated by Pic2, a Mitochondrial Carrier Family Protein. J. Biol. Chem..

[96] Furumoto T. (2016). Pyruvate transport systems in organelles: Future directions in C4 biology research. Curr. Opin. Plant. Biol..

[97] Herzig S., Raemy E., Montessuit S., Veuthey J.L., Zamboni N., Westermann B., Kunji E.R., Martinou J.C. (2012). Identification and functional expression of the mitochondrial pyruvate carrier. Science.

[98] Taochy C., Gaillard I., Ipotesi E., Oomen R., Leonhardt N., Zimmermann S., Peltier J.B., Szponarski W., Simonneau T., Sentenac H. (2015). The Arabidopsis root stele transporter NPF2.3 contributes to nitrate translocation to shoots under salt stress. Plant J..

[99] Marreddy R.K., Pinto J.P., Wolters J.C., Geertsma E.R., Fusetti F., Permentier H.P., Kuipers O.P., Kok J., Poolman B. (2011). The response of Lactococcus lactis to membrane protein production. PLoS ONE.

[100] Monné M., Robinson A.J., Boes C., Harbour M.E., Fearnley I.M., Kunji E.R. (2007). The mimivirus genome encodes a mitochondrial carrier that transports dATP and dTTP. J. Virol..

[101] Janvilisri T., Venter H., Shahi S., Reuter G., Balakrishnan L., van Veen H.W. (2003). Sterol transport by the human breast cancer resistance protein (ABCG2) expressed in Lactococcus lactis. J. Biol. Chem..

[102] Stribny J., Thines L., Deschamps A., Goffin P., Morsomme P. (2020). The human Golgi protein TMEM165 transports calcium and manganese in yeast and bacterial cells. J. Biol. Chem..

[103] Mifsud J., Ravaud S., Krammer E.M., Chipot C., Kunji E.R., Pebay-Peyroula E., Dehez F. (2013). The substrate specificity of the human ADP/ATP carrier AAC1. Mol. Membr. Biol..

[104] King M.S., Thompson K., Hopton S., He L., Kunji E.R.S., Taylor R.W., Ortiz-Gonzalez X.R. (2018). Expanding the phenotype of de novo SLC25A4-linked mitochondrial disease to include mild myopathy. Neurol. Genet..

[105] Zhang Y., Tian D., Matsuyama H., Hamazaki T., Shiratsuchi T., Terada N., Hook D.J., Walters M.A., Georg G.I., Hawkinson J.E. (2016). Human Adenine Nucleotide Translocase (ANT) Modulators Identified by High-Throughput Screening of Transgenic Yeast. J. Biomol. Screen..

[106] Boulet A., Vest K.E., Maynard M.K., Gammon M.G., Russell A.C., Mathews A.T., Cole S.E., Zhu X., Phillips C.B., Kwong J.Q. (2018). The mammalian phosphate carrier SLC25A3 is a mitochondrial copper transporter required for cytochrome c oxidase biogenesis. J. Biol. Chem..

[107] Schleifer K.H., Kraus J., Dvorak C., Kilpper-Bälz R., Collins M.D., Fischer W. (1985). Transfer of Streptococcus lactis and related streptococci to the genus Lactococcus gen. nov. Syst. Appl. Microbiol..

[108] Marreddy R.K., Geertsma E.R., Permentier H.P., Pinto J.P., Kok J., Poolman B. (2010). Amino acid accumulation limits the overexpression of proteins in Lactococcus lactis. PLoS ONE.

[109] Shilling R., Federici L., Walas F., Venter H., Velamakanni S., Woebking B., Balakrishnan L., Luisi B., van Veen H.W. (2005). A critical role of a carboxylate in proton conduction by the ATP-binding cassette multidrug transporter LmrA. FASEB J..

[110] Agboh K., Lau C.H.F., Khoo Y.S.K., Singh H., Raturi S., Nair A.V., Howard J., Chiapello M., Feret R., Deery M.J. (2018). Powering the ABC multidrug exporter LmrA: How nucleotides embrace the ion-motive force. Sci. Adv..

[111] Hellmich U.A., Glaubitz C. (2009). NMR and EPR studies of membrane transporters. Biol. Chem..

[112] Hellmich U.A., Lyubenova S., Kaltenborn E., Doshi R., van Veen H.W., Prisner T.F., Glaubitz C. (2012). Probing the ATP hydrolysis cycle of the ABC multidrug transporter LmrA by pulsed EPR spectroscopy. J. Am. Chem. Soc..

[113] Hellmich U.A., Mönkemeyer L., Velamakanni S., van Veen H.W., Glaubitz C. (2015). Effects of nucleotide binding to LmrA: A combined MAS-NMR and solution NMR study. Biochim. Biophys. Acta.

[114] Erkens G.B., Berntsson R.P., Fulyani F., Majsnerowska M., Vujičić-Žagar A., Ter Beek J., Poolman B., Slotboom D.J. (2011). The structural basis of modularity in ECF-type ABC transporters. Nat. Struct. Mol. Biol..

[115] Swier L.J., Guskov A., Slotboom D.J. (2016). Structural insight in the toppling mechanism of an energy-coupling factor transporter. Nat. Commun..

[116] Woebking B., Velamakanni S., Federici L., Seeger M.A., Murakami S., van Veen H.W. (2008). Functional role of transmembrane helix 6 in drug binding and transport by the ABC transporter MsbA. Biochemistry.

[117] Doshi R., Woebking B., van Veen H.W. (2010). Dissection of the conformational cycle of the multidrug/lipidA ABC exporter MsbA. Proteins.

[118] Doshi R., van Veen H.W. (2013). Substrate Binding Stabilizes a Pre-translocation Intermediate in the ATP-binding Cassette Transport Protein MsbA. J. Biol. Chem..

[119] Trip H., Mulder N.L., Lolkema J.S. (2013). Cloning, expression, and functional characterization of secondary amino acid transporters of Lactococcus lactis. J. Bacteriol..

[120] Ter Horst R., Lolkema J.S. (2010). Rapid screening of membrane topology of secondary transport proteins. Biochim. Biophys. Acta.

[121] Halestrap A.P. (1978). Stimulation of pyruvate transport in metabolizing mitochondria through changes in the transmembrane pH gradient induced by glucagon treatment of rats. Biochem. J..

[122] Miras S., Salvi D., Ferro M., Grunwald D., Garin J., Joyard J., Rolland N. (2002). Non-canonical transit peptide for import into the chloroplast. J. Biol. Chem..

[123] Kühlbrandt W. (2004). Biology, structure and mechanism of P-type ATPases. Nat. Rev. Mol. Cell Biol..

[124] Catty P., Boutigny S., Miras R., Joyard J., Rolland N., Seigneurin-Berny D. (2011). Biochemical characterization of AtHMA6/PAA1, a chloroplast envelope Cu(I)-ATPase. J. Biol. Chem..

[125] Neuhaus H.E., Thom E., Möhlmann T., Steup M., Kampfenkel K. (1997). Characterization of a novel eukaryotic ATP/ADP translocator located in the plastid envelope of *Arabidopsis thaliana* L.. Plant J..

[126] Tjaden J., Schwöppe C., Möhlmann T., Quick P.W., Neuhaus H.E. (1998). Expression of a plastidic ATP/ADP transporter gene in Escherichia coli leads to a functional adenine nucleotide transport system in the bacterial cytoplasmic membrane. J. Biol. Chem..

[127] Hostetler K.Y., Van den Bosch H., Van Deenen L.L. (1971). Biosynthesis of cardiolipin in liver mitochondria. Biochim. Biophys. Acta.

[128] Block M.A., Douce R., Joyard J., Rolland N. (2007). Chloroplast envelope membranes: A dynamic interface between plastids and the cytosol. Photosynth. Res..

[129] Wolters J.C., Berntsson R.P., Gul N., Karasawa A., Thunnissen A.M., Slotboom D.J., Poolman B. (2010). Ligand binding and crystal structures of the substrate-binding domain of the ABC transporter OpuA. PLoS ONE.

[130] Swier L.J., Monjas L., Guskov A., de Voogd A.R., Erkens G.B., Slotboom D.J., Hirsch A.K. (2015). Structure-based design of potent small-molecule binders to the S-component of the ECF transporter for thiamine. ChemBioChem..

[131] Sikkema H.R., van den Noort M., Rheinberger J., de Boer M., Krepel S.T., Schuurman-Wolters G.K., Paulino C., Poolman B. (2020). Gating by ionic strength and safety check by cyclic-di-AMP in the ABC transporter OpuA. Sci. Adv..

[132] Jäger F., Lamy A., Guerini N., Sun W.S., Berntsson R.P.A. (2020). Structure of the enterococcal T4SS protein PrgL reveals unique dimerization interface in the VirB8 protein family. bioRxiv.

[133] Focht D., Neumann C., Lyons J., Eguskiza Bilbao A., Blunck R., Malinauskaite L., Schwarz I.O., Javitch J.A., Quick M., Nissen P. (2021). A non-helical region in transmembrane helix 6 of hydrophobic amino acid transporter MhsT mediates substrate recognition. EMBO J..

[134] Ploetz E., Schuurman-Wolters G.K., Zijlstra N., Jager A.W., Griffith D.A., Guskov A., Gouridis G., Poolman B., Cordes T. (2021). Structural and biophysical characterization of the tandem substrate-binding domains of the ABC importer GlnPQ. Open Biol..

[135] Harborne S.P., Ruprecht J.J., Kunji E.R. (2015). Calcium-induced conformational changes in the regulatory domain of the human mitochondrial ATP-Mg/Pi carrier. Biochim. Biophys. Acta.

[136] Januliene D., Moeller A. (2021). Single-Particle Cryo-EM of Membrane Proteins. Methods Mol. Biol..

[137] Berntsson R.P., Alia Oktaviani N., Fusetti F., Thunnissen A.M., Poolman B., Slotboom D.J. (2009). Selenomethionine incorporation in proteins expressed in Lactococcus lactis. Protein Sci..

[138] Martens C. (2020). Membrane Protein Production in Lactococcus lactis for Structural Studies. Methods Mol. Biol..

